# One Health assessment of fascioliasis and schistosomiasis in humans, livestock, and snail vectors in peri-urban Rwanda

**DOI:** 10.14202/vetworld.2026.1368-1386

**Published:** 2026-03-28

**Authors:** Jean Bosco Ntivuguruzwa, Pie Ntampaka, Edmond Twagirayezu, Serene Isingizwe, Jacques Habumugisha, Valens Mbonyinshuti, Jean Damascene Bariyanga, Margaret Tumusiime, Viateur Manirarora, Eric Sibomana, Jean Bosco Mbonigaba, Ephrasia A. Hugho, AbdulHamid S. Lukambagire, Martin Ntawubizi

**Affiliations:** 1 School of Veterinary Medicine, University of Rwanda, Nyagatare Campus, Rwanda; 2 Center of Excellence in Biodiversity and Natural Resource Management, University of Rwanda, Huye Campus, Rwanda; 3 Neglected Tropical Disease and Other Parasitic Disease Unit, Rwanda Biomedical Centre, Kigali, Rwanda; 4 Kilimanjaro Clinical Research Institute, Moshi, Tanzania; 5 School of Public Health, KCMC University, Moshi, Tanzania

**Keywords:** fascioliasis, livestock, One Health, peri-urban Rwanda, schistosomiasis, snail vectors, trematode infections, wetlands

## Abstract

**Background and Aim:**

Fascioliasis and schistosomiasis are significant snail-borne trematode infections that affect humans and livestock in tropical regions, leading to notable public health and economic impacts. In Rwanda, previous studies have examined these diseases separately in humans or animals, but integrated surveillance encompassing humans, livestock, and snail vectors is missing. This study aimed to assess the prevalence, transmission patterns, and related risk factors of fascioliasis and schistosomiasis in humans, livestock, and freshwater snails, using a One Health approach in peri-urban districts of Kigali City, Rwanda.

**Materials and Methods:**

A cross-sectional One Health study was conducted from November 2023 to July 2024 in Gasabo and Kicukiro districts. Fecal samples were collected from livestock at farms (n = 120) and at the Nyabugogo abattoir (n = 150), and examined using sedimentation and postmortem inspection techniques. Human samples (n = 120) were analyzed with the Flukefinder® technique and direct microscopy. Freshwater snails (n = 222) were collected from 15 wetlands and rivers using a 500 μm mesh kick-net, identified morphologically, and examined for cercarial shedding. Water physicochemical parameters were measured at each sampling site. Logistic regression and descriptive statistics were employed to evaluate prevalence and risk factors.

**Results:**

The prevalence of fascioliasis in farm livestock was 40.8%, whereas schistosomiasis prevalence was 7.5%. At the abattoir, fascioliasis prevalence was 33.3%, and no schistosomiasis lesions were detected. Animals with lower body condition scores had significantly higher odds of *Fasciola* infection (OR = 43.33, p = 0.002). All *Schistosoma*-positive animals originated from Masaka sector (p < 0.0001). Among 222 snails collected, 1.4% shed cercariae, including *Biomphalaria pfeifferi*, *Biomphalaria sudanica*, and *Lymnaea natalensis*. Environmental parameters were generally favorable for snail survival, although only temperature showed significant association with cercarial shedding. No human infections were detected, likely due to low egg shedding or sampling limitations.

**Conclusion:**

This initial integrated One Health study in peri-urban Rwanda reveals active transmission of fascioliasis and schistosomiasis in livestock and snail vectors, suggesting a potential risk for human infection despite no current human cases. The detection of infected snails near livestock farms and wetlands indicates transmission hotspots that call for coordinated control efforts. Recommendations include regular livestock deworming, snail control, environmental management, and community education programs. Further longitudinal and molecular studies are necessary to better understand transmission dynamics and to support national One Health surveillance for neglected tropical diseases.

## INTRODUCTION

Fascioliasis and schistosomiasis are significant neglected tropical diseases (NTDs) that affect both humans and livestock globally, causing major socioeconomic and public health issues [1, 2]. These zoonotic parasitic infections are caused by snail-borne trematodes and remain a continuous threat to global health, especially in tropical and subtropical regions [3–5]. It is estimated that around 250 million people are affected by schistosomiasis, while nearly 180 million are affected by fascioliasis worldwide [3, 6]. The transmission of fascioliasis and schistosomiasis relies on aquatic snails from the families *Lymnaeidae* and *Planorbidae*, which serve as intermediate hosts during the parasite’s life cycle [7]. The survival and development of these parasites are heavily influenced by environmental factors such as temperature, humidity, and snail biology, which determine where the infections spread [8]. Fascioliasis, caused by *Fasciola hepatica* and *Fasciola gigantica*, is a major parasitic disease impacting the veterinary and agricultural sectors, especially in tropical areas [9]. Schistosomiasis, caused by species of *Schistosoma*, is also widespread in tropical and subtropical regions, and the development of parasites within snail hosts is affected by temperature, snail age, size, and nutritional status [10]. Climate change and ecological alterations have increased transmission risks by expanding suitable habitats for intermediate hosts and extending their reproductive cycles. Agricultural intensification, dam building, irrigation, and shifts in rainfall patterns have further contributed to the rising prevalence of these diseases [10, 11].

Snail-borne parasitic diseases impose a significant burden on livestock production and public health [3, 4, 6]. Fascioliasis and schistosomiasis decrease livestock productivity by reducing milk and meat output, fertility, and increasing mortality, leading to considerable economic losses. Internal parasitism caused by these trematodes results in poor weight gain, reproductive problems, and diminished working capacity in animals. For example, fascioliasis was reported to cause liver condemnation in cattle in Kilimanjaro, Tanzania, resulting in annual losses of USD 18,000 [12]. In Rwanda, only limited farm-level studies on fascioliasis in ruminants have been documented [13]. Schistosomiasis is also linked with substantial disability, with disability-adjusted life years estimated to exceed 2,355 in 2017 [14]. Additionally, liver condemnation due to fascioliasis at the Nyabugogo abattoir in Kigali caused economic losses of USD 8,932.40, illustrating the economic impact of these infections on the livestock sector [15].

The public health significance of these trematode infections is high because human infection occurs through contact with contaminated freshwater, either by ingesting infective stages or through skin penetration in the case of *Schistosoma* spp., making wetlands and marshlands high-risk environments [10]. Fascioliasis can cause severe liver complications, including biliary obstruction, liver abscesses, and cholangiocarcinoma [16], while chronic schistosomiasis in children may lead to liver fibrosis, intestinal damage, bladder cancer, and impaired growth and cognitive development [17]. Worldwide, more than 200 million people are infected with schistosomiasis, and around 2.4 million cases of fascioliasis are reported each year [18]. These diseases mainly impact low-income populations and contribute to poverty, malnutrition, and decreased productivity [19, 20]. Effective prevention depends on improved sanitation, access to safe water, health education, and targeted chemotherapy programs [18].

**Figure 1:**
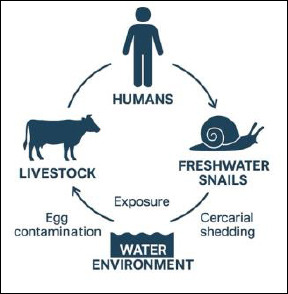
One Health concept of fascioliasis and schistosomiasis. [Source: This figure was generated using artificial intelligence (ChatGPT 5.3)].

Because the epidemiology of fascioliasis and schistosomiasis involves interactions among humans, livestock, freshwater snails, and environmental factors, control strategies need a One Health approach that combines veterinary, medical, and environmental surveillance. This approach allows for the simultaneous monitoring of snail distribution, human infection, and livestock infection, enabling coordinated interventions such as strategic deworming, water sanitation and hygiene (WASH), and mass drug administration. However, reinfection from contaminated environments and emerging drug resistance remain significant challenges [21]. The figure below illustrates the One Health concept of *Fasciola* spp. and *Schistosoma* spp. (Figure 1).

Although many studies have been conducted in East Africa, data on the combined epidemiology of fascioliasis and schistosomiasis in Rwanda are still limited, especially in peri-urban areas where livestock, humans, and freshwater snails often share wetlands and grazing lands [4, 22–24]. Past research in Rwanda has shown human schistosomiasis prevalence ranging from 2.7% to 67% [25–29] and fascioliasis in cattle mainly through abattoir surveys [13, 30], but these studies were done separately without assessing humans, livestock, and snail hosts at the same time. The lack of integrated studies involving livestock, snails, and humans restricts understanding of how the diseases spread and hampers efforts to develop effective One Health control strategies in high-risk areas like rice-growing wetlands. Furthermore, peri-urban districts of Kigali City have been underrepresented in previous epidemiological research, despite the strong connection between livestock farming, irrigation, and freshwater bodies.

Therefore, this integrated One Health study was conducted to determine the prevalence, transmission dynamics, and risk factors associated with fascioliasis and schistosomiasis in humans, livestock, and freshwater snails in peri-urban areas of the Gasabo and Kicukiro districts of Kigali City, Rwanda. The study aimed to provide the first simultaneous cross-species assessment in the country by combining parasitological, malacological, and environmental investigations. Additionally, it evaluated the distribution and infectivity of freshwater snail vectors and examined ecological and host-related factors that contribute to transmission, thereby offering baseline data to support the development of coordinated One Health surveillance and control programs for snail-borne trematode infections in Rwanda.

## MATERIALS AND METHODS

### Ethical approval

This study was conducted in full compliance with international ethical standards for research involving human participants and animals, including the Declaration of Helsinki (for human subjects), the World Organization for Animal Health (WOAH/OIE) Terrestrial Animal Health Code (for animal welfare), and the principles of the 3Rs (Replacement, Reduction, Refinement) for animal use.

#### Human participants component

The human research aspects, including community-based sampling (e.g., stool and/or urine collection for parasitological diagnosis), questionnaires on risk factors, demographic data, and informed consent processes, were reviewed and approved by the Institutional Review Board (IRB) of the College of Medicine and Health Sciences (CMHS) under the Rwanda National Ethics Committee (RNEC) (approval reference : No 176/CMHS IRB / 2023 dated [March 2023] submitted via researchcenter@ur.ac.rw, and the Rwanda Biomedical Centre: Ref No 10/RBC/2024.

Written informed consent was obtained from all adult participants prior to enrollment. For participants under 18 years of age (e.g., school-aged children in prevalence surveys), assent was obtained from the child, and written informed consent was provided by a parent or legal guardian. Information sheets and consent forms were available in Kinyarwanda and English, explaining the study purpose, procedures, voluntary nature, right to withdraw at any time without penalty, potential risks (minimal, non-invasive sampling), benefits (e.g., free diagnosis and treatment referral for positive cases per national/WHO guidelines), and confidentiality measures. Participants were assured that data would be anonymized and used only for research purposes. No incentives beyond standard compensation for travel/time were provided. Positive cases of schistosomiasis or other parasites were referred for treatment (e.g., praziquantel for schistosomiasis) following national protocols, with coordination from local health facilities.

Local permissions were obtained from relevant district authorities, community leaders, and/or schools prior to fieldwork.

#### Animal/livestock component

The animal-related aspects, including fecal sampling from livestock (e.g., cattle, sheep, goats), postmortem liver inspections at abattoirs, and collection of snail intermediate hosts, were reviewed and approved by the Institutional Animal Care and Use Committee (IACUC)/Animal Ethics Committee of the College of Agriculture, Animal Sciences and Veterinary Medicine (CAVM), University of Rwanda (approval reference: CAVM/65/2023, April 2023).

Sampling procedures adhered to humane, non-invasive or minimally invasive methods (e.g., rectal fecal collection without restraint stress, opportunistic abattoir sampling during routine slaughter under veterinary supervision). No live animals were euthanized solely for research purposes. Animal handling followed WOAH guidelines and national veterinary standards to minimize pain, distress, or suffering. Personnel involved were trained and competent in parasitological and veterinary procedures. Data from animals were anonymized by farm/abattoir identifiers.

#### Additional clearances and oversight

- Permissions for abattoir access and livestock sampling were obtained from the Ministry of Agriculture and Animal Resources (MINAGRI), district veterinary offices, and relevant abattoir managements.
- No conflicts of interest were declared by the investigators.
- All amendments to the protocol were submitted for re-approval where required.
- Data management ensured confidentiality, secure storage, and compliance with data protection principles.

### Study period and location

A cross-sectional One Health study was conducted from November 2023 to July 2024 to estimate the prevalence of fascioliasis and schistosomiasis in livestock at both farm and abattoir levels, as well as in symptomatic patients attending healthcare facilities within the study area.

### Study area and design

Livestock, snail, and human samples were collected simultaneously from the same ecological settings, allowing a cross-species assessment of fascioliasis and schistosomiasis transmission. This design is novel in Rwanda, where previous studies have largely focused on a single host species or isolated settings. In addition, physicochemical water parameters were measured at each sampling site, adding an ecological dimension that is rarely incorporated into regional NTD studies and enabling the evaluation of environmental factors associated with parasite transmission.

This One Health study was carried out in the peri-urban areas of Gasabo and Kicukiro districts in Kigali City, Rwanda. Kigali City is the fifth province of Rwanda and is located near the equator. Gasabo district is situated at latitude -1.8847 and longitude 30.13141. The district experiences a mean annual temperature of 20.9°C and receives approximately 908 mm of rainfall each year. Its landscape features high mountains with an average elevation of 1,800 meters, valleys, and over 30 wetlands. The main river system runs from Lake Muhazi to the Nyabugogo and Nyabarongo rivers, with other significant rivers including Rusumo and Buliza [31].

Kicukiro district is situated in the southeastern part of Kigali City at latitude -2.0039 and longitude 30.1470. Both Kicukiro and Gasabo districts have a tropical climate. In Kicukiro, the average annual temperature ranges from 15.2°C to 24.8°C, humidity ranges from 54% to 86%, annual rainfall varies from 13 to 186 mm, and wind speed averages approximately 10.5 km/h [32].

In Rwanda, part of the marshland is currently used for rice cultivation, representing 1.2% of the main crops grown in Gasabo district. Rice is primarily cultivated in low-lying marshy wetlands. For example, Kabuye marshland, which stretches across Nduba and Jabana sectors in Gasabo district, is used for rice production by smallholder cooperatives, including Cooperative de Riz de Kabuye (CORIKA) in Jabana sector and Cooperative pour la Promotion du Riz (COPRORIZ) in Nduba sector. Additionally, Rugende marshland, covering about 400 hectares across Gasabo, Kicukiro, and Rwamagana, has also been developed for rice production [33]. Freshwater bodies, farms, and health facilities included in this study are located near these marshlands.

**Figure 2:**
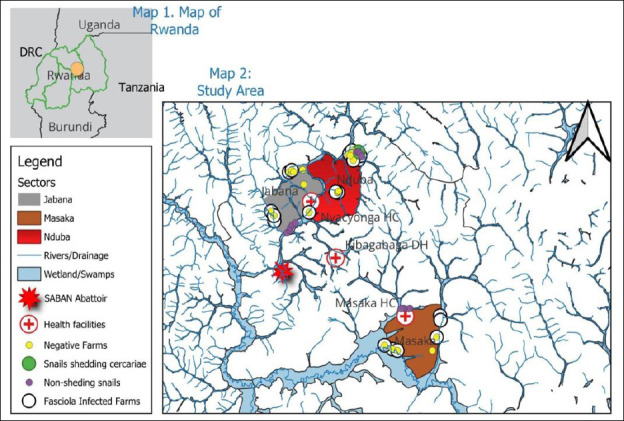
” Map 1. Map of Rwanda with neighboring countries; Map 2. Three sectors (Jabana, Masaka, and Nduba) of the study area with wetlands, rivers, and farms. This map was created using a free online QGIS Desktop version 3.34.0. The spatial data (shapefiles) used are freely available from DIVA-GIS at https://gadm.org/download_country.html.

The livestock component included both cattle and small ruminants (goats and sheep) and was conducted at farm and abattoir levels. The abattoir-based survey was performed at Société des Abattoirs de Nyabugogo (SABAN) abattoir in Kigali, Rwanda, a modern slaughter facility with a capacity of approximately 200 cattle per day. The farm-based component was carried out in Jabana and Nduba sectors of Gasabo district and Masaka sector of Kicukiro district. A total of 31 farms located near rice plantations, rivers, and water bodies were selected for the study (Figure 2). Human participants were recruited from three health facilities, namely Kibagabaga District Hospital, Masaka Health Center, and Nyacyonga Health Center, all serving peri-urban populations of Kigali City.

### Sample size determination

To ensure adequate statistical precision, sample sizes for humans, slaughtered animals, and live livestock were calculated using Cochran’s formula. Because no similar previous study had been conducted in the study area, an expected prevalence of 50% was assumed. A precision of 8% was used for slaughtered animals, whereas a precision of 9% was used for humans and live animals.

Sample size formula: n = Z²P(1 − P)/d²

For the abattoir component:

n = (1.96² × 0.5 [1 − 0.5])/(0.08)²= (3.8416 × 0.25)/0.0064= 0.9604/0.0064= 150.06 ≈ 150

At a 95% confidence level, Z was 1.96. With an expected prevalence of 50% and a precision of 8%, the minimum required sample size for slaughtered animals was 150. The selected abattoir receives cattle, goats, and sheep from more than 24 districts across the country. Accordingly, 100 cattle, 30 goats, and 20 sheep were sampled at the abattoir during three visits per week.

For humans and live animals:

n = (1.96² × 0.5 [1 − 0.5])/(0.09)²= (3.8416 × 0.25)/0.0081= 0.9604/0.0081= 118.56 ≈ 120

Therefore, the study ultimately included 120 human participants (40 from each health facility) and 120 farm animals from 31 farms. The farm animals consisted of cattle (n = 91), goats (n = 15), and sheep (n = 14) from peri-urban areas of Gasabo and Kicukiro districts. These two districts were chosen because of the presence of livestock farms, water bodies, and rice plantations, and because they had been underrepresented in previous studies. A stratified random sampling method was used to select patients attending the health facilities. In this study, four strata were considered: patients from the three health facilities, farm livestock, slaughtered livestock, and snails.

### Sampling strategy and study population

#### Livestock component

A purposive sampling method was employed to select three sectors with farms located near rice plantations and water bodies. Out of the 31 farms chosen, 9 were in Jabana, 6 in Masaka, and 15 in Nduba. From each sector, 40 animals were randomly selected (Table 1). Data on individual animal characteristics, including age, sex, origin, and breed (crossbred and Friesian), were recorded using dentition as previously described [34]. Additionally, deworming history and physiological status were documented through face-to-face interviews with farmers at each farm. During postmortem inspections at the abattoir, the mesenteric veins and livers were examined for adult *Schistosoma* and *Fasciola* flukes, respectively. All records were entered into Microsoft Excel 365 (Microsoft Office, WA, USA).

**Table 1:** Number of farms and number of animal species sampled in each sector of the integrated study.

Sectors	Number of farms	Number of cows	Number of goats	Number of sheep	Total animals
Jabana	9	28	7	5	40
Nduba	15	35	5	0	40
Masaka	6	28	3	9	40
Total	31	91	15	14	120

#### Human component

The objectives of the One Health study were explained to patients at the selected health facilities who showed symptoms indicative of trematode infection, such as abdominal pain, recurrent fever, bloody diarrhea, hematuria, and hepatomegaly, and who lived in the study area. Those who agreed to participate were enrolled. Individuals who declined participation and those who had received anthelmintic treatment within the past 2 weeks were excluded to reduce the chances of false-negative stool results.

### Collection and handling of samples

#### Snail samples

**Figure 3:**
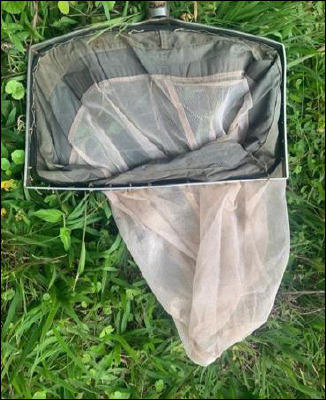
A 500 μm kick-net used during freshwater snail identification.

**Figure 4:**
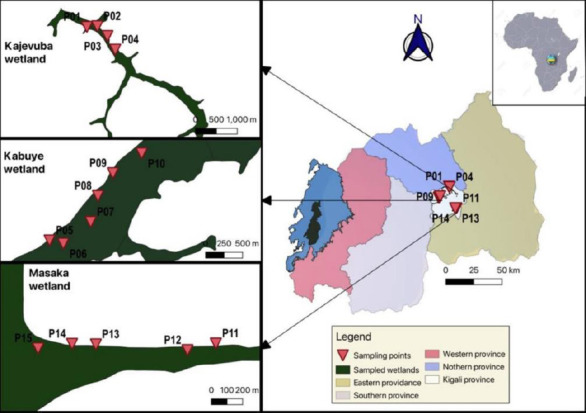
General map of the study area showing the malacological sampling sites in the different wetlands.

Snails were collected using a 500 μm mesh-size kick-net (Figure 3) from 15 purposively selected sites along the Nyabarongo River. At each site, samples were gathered over a 3 m² area within 5 min of kicking and sorted for at least 30 min. Three sites were located in Kajevuba wetland, six in Kabuye wetland, and five in Masaka wetland (Figure 4). Water parameters, including pH, temperature, electrical conductivity, sodium chloride, and redox potential, were measured at each site using an HQ40d multimeter equipped with specific probes. The IntelliCAL PHC101 probe (S/N: 160322568063) was used for pH, redox potential, and temperature, while the Hach CDC401 probe (S/N: 232572581105) was used for electrical conductivity and redox potential (Figure 4). Two investigators performed the snail collections. Collected snails were placed in labeled jars with water and transported within 24 h to the Rwanda Agriculture and Animal Resources Development Board (RAB, Rubirizi Station) laboratory for cercarial shedding. Three duplicate examinations were conducted for quality control. Waste materials generated during snail collection were placed in biohazard bags and transported to RAB for incineration.

#### Livestock samples

Fecal samples were collected directly from the rectum of animals at farms and at the abattoir by trained veterinarians wearing sterile gloves and masks. The samples were placed in a cool box containing ice packs and transported to the parasitology laboratory of the RAB for analysis using the rapid fecal sedimentation technique. Samples were stored at –20°C and analyzed on the same day by trained veterinary laboratory technicians. Animal data recorded included age, sex, sample origin, and breed. Deworming history and physiological status were recorded at the farm-level. Mesenteric veins and livers of slaughtered animals were examined during postmortem inspection for adult *Schistosoma* and *Fasciola* flukes, respectively. Data were recorded in Microsoft Excel.

#### Human samples

Each participant received two containers, one for stool and one for urine collection. Stool samples were immediately analyzed at healthcare facilities using the Flukefinder® (Provinos Ltd, Limburg, Netherlands) technique, while urine samples were examined through direct wet mount microscopy. Additional demographic information, including age, sex, education level, marital status, and economic activity, was gathered via face-to-face interviews with a structured questionnaire. The questionnaire was pretested on five participants who were later excluded from the study, and it was revised based on their feedback.

### Processing of diagnostic techniques

#### Fecal sedimentation (livestock)

Animal fecal samples were analyzed at the parasitology laboratory of the RAB as previously described [35]. A 1:20 fecal suspension was prepared by mixing 10 g of feces with 200 mL of tap water in a beaker. The mixture was filtered through a strainer into another beaker and allowed to sediment for 10 min. After discarding 70% of the supernatant, the sediment was resuspended three times in 200 mL of water, each time allowing 10 min for sedimentation. After the final sedimentation step, 90% of the supernatant was discarded, and the sediment was transferred to a Petri dish. One drop of 1% methylene blue (Loba Chemie Pvt Ltd, Mumbai, India) was added to stain fecal debris deep blue while leaving trematode eggs unstained. Eggs were examined morphologically under a light microscope at 10× magnification, and a sample was considered positive when at least one egg was detected. Two trained laboratory technicians independently examined each slide. *Fasciola* eggs were identified as large, yellow-brown, oval, and operculated, measuring 130–150 × 60–90 μm. *Schistosoma* eggs were yellowish and non-operculated; *Schistosoma mansoni* eggs have a lateral spine, *Schistosoma haematobium* eggs have a terminal spine, and *S. japonicum* eggs have a small lateral knob. Parasites were examined using 10× and 40× microscopy (Olympus, Japan) [35].

#### Postmortem examination (abattoir)

Livers and mesenteric veins of slaughtered animals were examined during postmortem inspection for the macroscopic presence of adult *Fasciola* and *Schistosoma* flukes and their associated lesions. Fascioliasis was diagnosed by the presence of blackish adult *Fasciola* in the bile ducts, while schistosomiasis was identified by the presence of whitish adult *Schistosoma* and congestion of the mesenteric veins [36].

#### Flukefinder® (Provinos Botvanger ltd, German) and direct microscopy (humans)

The Flukefinder® technique was employed for human stool examination as previously described [37, 38]. Flukefinder® exhibits high sensitivity for detecting *Fasciola* at moderate egg burdens but has reduced sensitivity in light infections [38]. Briefly, 2 g of stool sediment was mixed with 30 mL of water and poured into the top section of the Flukefinder® apparatus. The sample was flushed four times with moderate-force tap water while shaking the apparatus by hand to expel water through the screens. The top unit was then separated, and the debris in the upper section was discarded. The lower section was inverted, and the eggs and remaining debris were backwashed into a cup using a strong stream of water from a squeeze bottle. The recovered suspension was transferred into 10 mL of water and left to settle for 4 min. The supernatant was discarded, and the tube was refilled with 10 mL of water. This step was repeated, after which the suspension was allowed to settle for 2 min. The final supernatant was discarded, and the sediment was swirled and quickly poured into a shallow scribed dish. An additional squirt of water was used to rinse the tube contents into the dish. A drop of methylene blue was added, and the sample was examined under a microscope using the 10× objective lens. A sample was considered positive if at least one egg was detected. All samples were processed immediately after collection.

The wet mount technique for urine examination was performed as previously described [35]. Briefly, 10 mL of urine was centrifuged at 1500-2000 rpm (around 250-400 × *g*) for 5 min to concentrate eggs. The supernatant was discarded, leaving about 0.5 mL of sediment, which was mixed with a drop of methylene blue. One drop of this mixture was placed on a clean slide, covered with a coverslip, and examined under a light microscope with 10× and 40× objectives. The sample was considered positive if at least one egg was seen. All samples were processed immediately after collection.

#### Cercarial shedding and identification of snails

After collection, snails were transported to the parasitology laboratory at the RAB for identification using a field guide to African freshwater snails (East African species, second edition) [39]. Cercarial shedding was stimulated by placing individual snails, known to be intermediate hosts, into clean containers with dechlorinated water and exposing them to bright 60 W light for 2.5 h, repeated in three 45-min intervals. Water temperature was maintained between 25°C and 28°C, which is considered optimal for cercarial emergence. After exposure, the water was examined under a microscope at 40× magnification. Snails that did not shed cercariae after two consecutive trials were dissected to detect prepatent infections. Identification of snails was based on shell morphology, including shell shape, spire height, aperture form, and ornamentation [39]. Cercariae were identified by tail structure, body shape, sucker arrangement, and pigmented eye spots. Future studies should include polymerase chain reaction or loop-mediated isothermal amplification for molecular confirmation of snail and cercarial species. Spearman’s correlation analysis was used to assess the relationships between water parameters and snail abundance, as well as between water parameters and cercarial shedding.

#### Physicochemical analysis of water

Water physicochemical parameters were measured using an HQ40d multimeter with specific probes to record temperature (°C), pH, NaCl, and electrical conductivity (μS/cm). Measurements were taken at a depth of 10–20 cm during morning hours (08:00–10:00) to minimize diurnal variation. Three replicate readings were recorded at each site. Before use, the probes were calibrated according to the manufacturer’s instructions using standard buffer and conductivity solutions. Acceptable ranges followed standard freshwater guidelines: temperature, 20°C–30°C; pH, 6.5–8.5; and conductivity, 50–1500 μS/cm.

#### Data management and statistical analysis

Data on the prevalence of fascioliasis and schistosomiasis in livestock (cattle, goats, and sheep) and humans were analyzed using appropriate statistical methods. Data cleaning was performed before analysis. Results from parasitological and postmortem investigations were entered into Microsoft Excel 365. Prevalence was calculated by dividing the number of infected individuals by the total study population and multiplying by 100. The same process was used to determine cercarial shedding rates.

Descriptive analysis, including proportions and 95% confidence interval, was performed for seven explanatory variables related to *Fasciola* and *Schistosoma* infections in farm livestock. Complete-case analysis was used to handle missing data. Univariate associations between explanatory variables and infection status were assessed with generalized linear models with binomial distribution and likelihood ratio tests. Variables with p < 0.25 were further examined for multicollinearity using Cramer’s V test. When collinearity was detected (Cramer’s V > 0.6), the biologically more plausible variable was retained for subsequent analysis. Retained variables were included in multivariable generalized linear models using backward and forward elimination to identify factors significantly associated with *Fasciola* or *Schistosoma* infection. Possible interactions between retained variables were also evaluated. Model goodness-of-fit was assessed using receiver operating characteristic analysis and area under the curve values. A model was considered acceptable when the area under the curve (AUC) was >0.5. Statistical analysis was performed with R software version 4.5.1 [40], at a 5% significance level with the “ROCR,” “pROC,” and “MASS” packages. The association between snail abundance and physicochemical water parameters was tested using Spearman’s correlation.

## RESULTS

### Integrated One Health findings

This comprehensive One Health analysis, which combines data from snails, livestock, and humans, highlighted localized transmission hotspots and emphasized the importance of integrated surveillance.

### Coprological prevalence of fascioliasis and schistosomiasis among slaughtered livestock

Out of 100 slaughtered cattle examined, 36.0% (95% CI: 25.6–45.4) tested positive for *Fasciola* spp., followed by goats at 30.0% (95% CI: 13.6–46.4) and sheep at 25.0% (95% CI: 6.0–44.0). Female animals showed a higher positivity rate for *Fasciola* spp. (39.4%, 95% CI: 27.6–51.2) compared to males (28.6%, 95% CI: 18.9–38.2). Younger animals (≤2 years for cattle and ≤8 months for small ruminants) had a higher positivity rate (36.8%, 95% CI: 25.3–48.2) than adults (>2 years for cattle and >8 months for small ruminants), which had a positivity rate of 30.5%. Crossbred animals showed a higher positivity rate (37.0%, 95% CI: 26.5–47.6) than local breeds (29.9%). Animals slaughtered from the Northern Province had the highest positivity rate for *Fasciola* spp. (42.4%, 95% CI: 29.8–55.0), followed by those from the Southern Province (40.0%) and Eastern Province (26.4%, 95% CI: 16.2–36.6). None of the slaughtered animals tested positive for *Schistosoma* spp. (Table 2).

**Table 2:** Simple univariable logistic regression analysis of *Fasciola* spp. prevalence and individual animal risk factors: Species, sex, breed, age, and province of origin.

Variable	Category	Tested	*Fasciola* spp. positive, n (%)	95% Confidence interval
Species	Cattle	100	36 (36.0)	25.6–45.4
	Goat	30	9 (30.0)	13.6–46.4
	Sheep	20	5 (25.0)	6.0–44.0
	Total	150	50 (33.3)	25.8–40.9
Sex	Male	84	24 (28.6)	18.9–38.2
	Female	66	26 (39.4)	27.6–51.2
	Total	150	50 (33.3)	25.8–40.9
Age	Young ≤2 years and 8 months of age	68	25 (36.8)	25.3–48.2
	Adult age >2 years and 8 months	82	25 (30.5)	20.5–40.5
	Total	150	50 (33.3)	25.8–40.9
Breed	Local	67	20 (29.9)	18.9–40.8
	Friesian	2	0 (0.0)	0.0–0.0
	Crossbreed	81	30 (37.0)	26.5–47.6
	Total	150	50 (33.3)	25.8–40.9
Provinces	Eastern	72	19 (26.4)	16.2–36.6
	Northern	59	25 (42.4)	29.8–55.0
	Southern	15	6 (40.0)	15.2–64.8
	Western	4	0 (0.0)	0.0–0.0
	Total	150	50 (33.3)	25.8–40.9

### Prevalence of Fasciola and Schistosoma in *slaughtered* animals using the meat inspection technique

*Fasciola* trematodes were macroscopically present in 36.0% of cattle (36/100, 95% CI: 26.6–45.4), 16.6% of goats (9/30, 95% CI: 13.6–46.4), and 25.0% of sheep (5/20, 95% CI: 6.0–44.0). Lesions specific to schistosomiasis were not documented in this study.

### Coprological prevalence of fascioliasis and schistosomiasis among farm livestock

Of the 31 farms included in the study, 22 (70.9%, 95% CI: 54.9–86.9) tested positive for *Fasciola* eggs, while only 2 (6.5%, 95% CI: 0.0–15.1) tested positive for *Schistosoma* eggs. A total of 120 fecal samples were collected from Jabana, Masaka, and Nduba sectors (40 samples per sector). The overall prevalence of *Fasciola* spp. was 40.8% (49/120, 95% CI: 32.0–49.6), and the prevalence of *Schistosoma* spp. was 7.5% (9/120, 95% CI: 2.8–12.2). One animal (0.8%) tested positive for both *Fasciola* and *Schistosoma*. The prevalence of *Fasciola* infection was highest in Nduba and Jabana sectors, both at 45.0% (18/40, 95% CI: 29.6–60.4), compared to 32.5% (13/40, 95% CI: 18.0–47.0) in Masaka sector. Notably, all *Schistosoma*-positive cases were found on farms 25 and 26 in Masaka sector, resulting in a sectoral prevalence of 22.5% (9/40, 95% CI: 9.6–35.4) (Table 3).

**Table 3:** Proportion of *Fasciola* and *Schistosoma* spp. detection cases per sector and animal species.

Sector	No. of farms (N), detection (n/N, %), 95% CI	Cattle (N), detected (n/N, %), 95% CI	Goats (N), detected (n/N, %), 95% CI	Sheep (N), detected (n/N, %), 95% CI	Total animals (N), detected (n/N, %), 95% CI
Jabana	9 (6/9, 66.6), 35.9–97.5	28 (14/28, 50.0), 31.5–68.5	7 (2/7, 28.6), 0.0–15.1	5 (2/5, 40.0), 0.0–82.9	40 (24/40, 60.0), 44.8–75.2
Nduba	15 (10/15, 66.6), 42.8–90.5	35 (17/35, 48.6), 32.0–65.1	5 (1/5, 20.0), 0.0–55.1	0 (0/0, 0.0), 0.0–0.0	40 (28/40, 70.0), 55.8–84.2
Masaka	6 (6/6, 100.0), 100.0–100.0	28 (9/28, 32.1), 14.9–49.4	3 (0/3, 0.0), 0.0–0.0	9 (6/9, 66.7), 35.9–97.5	40 (15/40, 37.5), 22.5–52.5
Total	31 (22/31, 70.9), 54.9–86.9	91 (40/91, 43.9), 33.8–54.2	15 (3/15, 20.0), 0.0–40.2	14 (8/14, 57.4), 31.2–83.1	120 (67/120, 55.8), 46.9–64.7

CI = Confidence interval.

### Univariate analysis of Fasciola and *Schistosoma* infections among farm livestock

Table 4 displays the univariate analyses of the seven explanatory variables. Of the seven initially assessed factors, only three variables—breed, animal species, and body condition score—had p < 0.25 in the univariate analysis for *Fasciola*, while only sector and breed met this criterion for *Schistosoma*. The variables “breed” and “animal species” were collinear (Cramer’s V = 0.713); therefore, breed was eliminated, and only animal species was further analyzed for *Fasciola* infection (Table 4).

**Table 4:** Descriptive and univariate analysis of *Fasciola* or *Schistosoma* infections among farm livestock with predictor factors.

Variables	*Fasciola* spp. positive n/N (%)	95% CI	p-value	*Schistosoma* spp. positive n/N (%)	95% CI	p-value
Sectors						
Nduba	18/40 (45.0)	29.6–60.4		0/40 (0.0)	0.0–0.0	
Jabana	18/40 (45.0)	29.6–60.4	0.42	0/40 (0.0)	0.0–0.0	0.0001
Masaka	13/40 (32.5)	18.0–47.0		9/40 (22.5)	9.6–35.4	
Total	49/120 (40.8)	32.0–49.6		9/120 (7.5)	2.8–12.2	
Body condition score (BCS)						
2.0	10/13 (76.9)	54.0–99.8		0/13 (0.0)	0.0–0.0	
3.0	38/93 (40.9)	30.9–50.9	0.0007	8/93 (8.6)	2.9–14.3	0.83
4.0	1/14 (7.1)	0.0–20.6		1/14 (7.1)	0.0–20.6	
Total	49/120 (40.8)	32.0–49.6		9/120 (7.5)	2.8–12.2	
Deworming status						
Never	2/5 (40.0)	0.0–82.9		1/5 (20.0)	0.0–55.1	
Don’t know	21/32 (65.6)	49.2–82.1		1/32 (3.1)	0.0–9.2	
1–3 months	16/56 (28.6)	18.6–44.1	0.0023	7/56 (12.5)	3.8–21.2	0.15
4–6 months	8/25 (32.0)	13.7–50.3		0/25 (0.0)	0.0–0.0	
10–12 months	2/2 (100.0)	100.0–100.0		0/2 (0.0)	0.0–0.0	
Total	49/120 (40.8)	32.0–49.6		9/120 (7.5)	2.8–12.2	
Breed						
Crossbreed	40/78 (51.3)	40.2–62.4		3/78 (3.8)	0.0–8.1	
Friesian	2/4 (50.0)	1.0–99.0	0.0013	0/4 (0.0)	0.0–0.0	0.096
Local	7/38 (18.4)	6.1–30.8		6/38 (15.8)	4.2–27.4	
Total	49/120 (40.8)	32.0–49.6		9/120 (7.5)	2.8–12.2	
Species						
Cattle	40/91 (43.9)	33.8–54.2	0.23	7/91 (7.7)	2.2–13.2	
Sheep	6/14 (42.9)	16.9–68.8		2/14 (14.3)	0.0–32.6	0.26
Goat	3/15 (20.0)	0.0–40.2		0/15 (0.0)	0.0–0.0	
Total	49/120 (40.8)	32.0–49.6		9/120 (7.5)	2.8–12.2	
Physiological status						
Heifer	3/7 (42.9)	—		1/7 (14.3)	—	0.66
Juvenile	1/2 (50.0)	—		0/2 (0.0)	—	
Lactating	43/107 (40.2)	—		8/107 (7.5)	—	
Pregnant	2/4 (50.0)	—		0/4 (0.0)	—	
Total	49/120 (40.8)	32.0–49.6		9/120 (7.5)	2.8–12.2	
Types of drugs used						
Albendazole	14/45 (31.1)	—	0.03	3/45 (6.7)	—	0.0032
Fluconix	1/7 (14.3)	—		4/7 (57.1)	—	
Ivermectin	0/2 (0.0)	—		0/2 (0.0)	—	
Levamisole	3/10 (30.0)	—		0/10 (0.0)	—	
Levamisole + Albendazole	5/10 (20.0)	—		0/10 (0.0)	—	
Not at all	1/5 (20.0)	—		1/5 (20.0)	—	
Don’t know	25/41 (60.9)	—		1/41 (2.4)	—	
Total	49/120 (40.8)	32.0–49.6		9/120 (7.5)	2.8–12.2	

CI = Confidence interval.

### Multivariate analysis of factors associated with fascioliasis among farm livestock

After the stepwise elimination process during multivariable logistic regression, only body condition score was retained as a significant factor associated with *Fasciola* infection, while only sector was retained for *Schistosoma* infection (Table 5). The odds of *Fasciola* infection were significantly higher in animals with lower body condition scores, and the model demonstrated acceptable goodness-of-fit (area under the curve = 0.644). For *Schistosoma* infection, animal origin (sector) had a significant effect on infection occurrence, with all infected animals (n = 9) coming from Masaka and none from Nduba or Jabana (Tables 4 and 5). The goodness-of-fit for the *Schistosoma* model was also good (AUC = 0.86).

**Table 5:** Multiple logistic regression analysis of factors associated with fascioliasis in farm livestock.

Variable	Level	Coefficient	Standard error	Odds ratio	95% CI of the odds ratio	Wald p-value	Likelihood ratio test p-value
*Fasciola*							
Body condition score (BCS)	4	Reference	—	—	—	—	0.0007
	3	2.195	1.059	8.98	1.13–71.57	0.038	
	2	3.769	1.229	43.33	3.89–481.82	0.002	
*Schistosoma*							
Sector	Masaka	Reference	—	—	—	—	<0.0001
	Jabana	–19.329	2803.418	<0.001	0–Inf	0.995	
	Nduba	–19.329	2803.418	<0.001	0–Inf	0.995	

CI = Confidence interval.

### Malacological survey and shedding of cercariae

**Figure 5:**
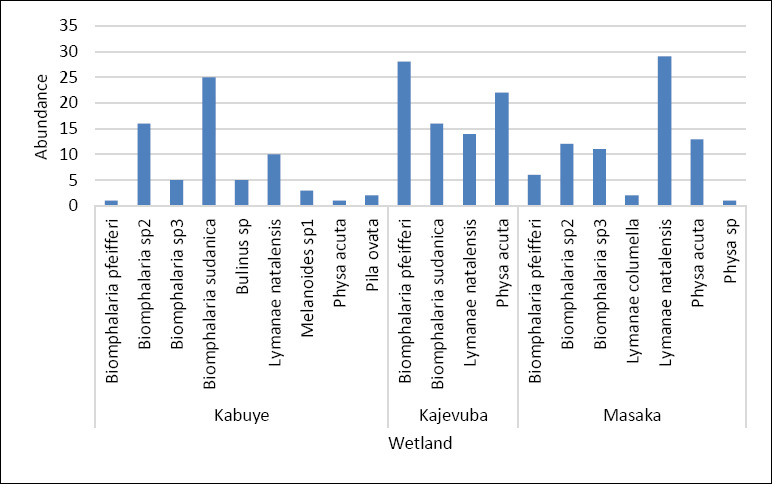
Snail vector abundance collected from three sampling freshwater points.

**Figure 6:**
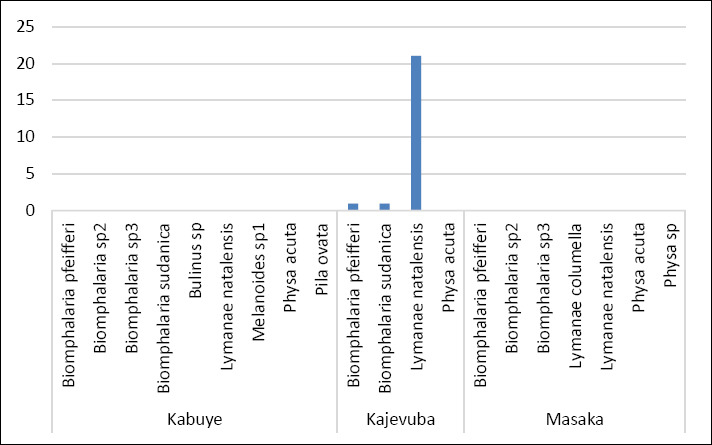
Different snail vectors and the corresponding shaded numbers of *Fasciola* and *Schistosoma* cercariae.

A total of 11 freshwater snail species were identified across the study sites, with three species observed shedding cercariae, marking the first report of cercarial shedding in these snails in Rwanda. Overall, 222 snails were collected from three rice-growing wetlands in peri-urban Kigali City: Kajevuba, Kabuye, and Masaka. The identified species included *Biomphalaria pfeifferi*, *Biomphalaria sudanica*, *Physa acuta*, *Lymnaea natalensis*, *Biomphalaria* sp. 2, *Biomphalaria* sp. 3, *Bulinus* spp., *Physa* spp., *Pila ovata*, *Melanoides* spp., and *Lymnaea columella*. The most abundant species were *Lymnaea natalensis* and *B. sudanica* (Figure 5). Only 1.4% (3/222) of the snails released cercariae: one *B. pfeifferi* released a *S. mansoni* cercaria, one *B. sudanica* released a *Fasciola* spp. cercaria, and one *L. natalensis* released 21 *Fasciola* spp. cercariae. All infected snails were found in Kajevuba wetland, Nduba sector (Figures 2 and 6). No snail released more than one type of cercaria.

**Figure 7:**
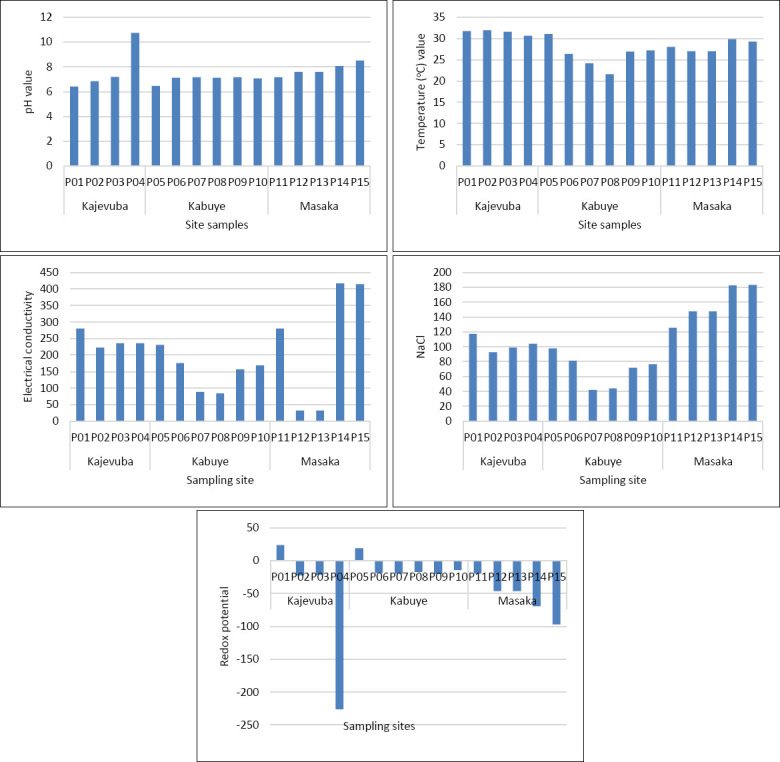
Water parameters including NaCl (mg L^-1^), pH, temperature (°C), electrical conductivity (μS cm^-1^), and redox potential (mV) across each sampling point.

The measured water parameters included NaCl, pH, electrical conductivity, temperature, and redox potential. The pH, temperature, electrical conductivity, redox potential, and salinity values peaked at point 1, showing clear variation in water chemistry across the sampling locations (Figure 7). However, these differences did not appear to influence snail abundance or cercarial presence. Redox potential was positive at points 1 and 5 and did not impact snail presence. Overall, the other water parameters showed minimal variation and no clear effect on snail occurrence. Spearman’s correlation indicated that pH, temperature, electrical conductivity, NaCl, and redox potential were all very weak predictors of cercarial count (|r| < 0.2); however, temperature was significantly linked to cercarial number (p = 0.0045), while all other predictors were not significantly associated (p > 0.05). Conversely, there was no strong linear relationship between snail species and water parameters, including redox potential (r = 0.165, p = 0.014), pH (r = 0.134, p = 0.047), NaCl (r = 0.091, p = 0.176), and temperature (r = 0.070, p = 0.300).

### Fascioliasis and schistosomiasis in participants attending health facilities

In contrast, no human cases were detected during the survey; however, this likely reflects underdiagnosis due to low or intermittent egg shedding rather than a true absence of infection.Most participants were aged 18–35 years [41.2% (59/119), 95% CI: 32.3–50.0], 56.3% (67/119, 95% CI: 47.4–65.2) were female, and 55.5% (66/119, 95% CI: 46.5–64.4) had primary education. Crop farming was the most common economic activity in the study area, accounting for 38.7% (46/119, 95% CI: 29.9–47.4). Livestock rearing represented 11.8% (14/119, 95% CI: 7.0–16.7). In this One Health study, none of the participants (0%) tested positive for schistosomiasis or fascioliasis by egg detection in stool or urine.

## DISCUSSION

### Overview of the One Health significance of fascioliasis and schistosomiasis

*Fasciola* and *Schistosoma* infections continue to pose significant threats to livestock productivity and public health globally, yet remain overlooked in many developing African countries [1, 41]. In this study, eggs of *Fasciola* and *Schistosoma* were found in fecal samples from cattle, sheep, and goats, and infected snail vectors were also identified. These results confirm the ongoing circulation of trematode infections at the livestock-snail-environment interface. Since definitive hosts, including humans and livestock, become infected through contact with contaminated water and ingestion of contaminated aquatic plants or food, understanding the epidemiology of these trematodiases is crucial for reducing infection risk and enhancing prevention strategies, especially through improved livestock management and decreased exposure to intermediate hosts [42].

### Fascioliasis in slaughtered livestock and its economic implications

The coprological findings from slaughtered animals in this One Health study showed that the prevalence of *Fasciola* infection was broadly similar to the 20% previously reported in Nyagatare district, Rwanda [13], and the 20.9% reported in slaughtered livestock in Tanzania [4]. However, the prevalence seen in this study was lower than that reported in the Imbo region of Burundi (47.7%) and at Lira municipal abattoir in northern Uganda (65.7%) [43]. Such variation across regions may be due to differences in environmental suitability for intermediate hosts, seasonal effects, and livestock management practices [24].

Postmortem examination further confirmed the presence of *Fasciola* trematodes in the livers of cattle (40.0%), goats (16.6%), and sheep (20.0%). These findings align with earlier reports from Rwanda showing that fascioliasis is a major cause of liver condemnation at slaughter. Previous postmortem studies documented infection rates of 41.1%, 90.0%, and 65.5% in cattle slaughtered in Bugesera, Gicumbi, and Rwamagana districts, respectively [30]. Another report from Nyabugogo abattoir indicated that 78.7% of liver condemnations were due to fascioliasis [15]. Collectively, these findings highlight the significance of fascioliasis as a source of direct economic loss through organ condemnation and decreased livestock value. Strengthening fascioliasis prevention and control in Rwandan livestock would likely improve animal productivity and indirectly reduce human exposure.

### Infection patterns and risk factors in farm livestock

In live farm animals, the overall prevalence of trematode infections (*Fasciola* and *Schistosoma*) was 47.5%, significantly higher than the 3.9% reported in Ethiopia [44]. The prevalence of *Fasciola* infection in this study (40.0%) exceeded that in Tanzania (21.7%) but was lower than in the Imbo region of Burundi (47.0%) [45]. Additionally, the prevalence of fascioliasis was greater than that of schistosomiasis (6.7%), aligning with findings from Ethiopia reporting prevalences of 20.5% for *Fasciola* and 6.3% for *Schistosoma* in various livestock species [46]. This variation may stem from biological and ecological differences between the two parasites. *Fasciola* has a broader host range, widespread snail intermediate hosts, and infective metacercariae that encyst on vegetation and are easily ingested during grazing. Conversely, *Schistosoma* requires more specific intermediate hosts, has a narrower host range, and depends on direct skin penetration by its short-lived infective stages, which reduces transmission opportunities in livestock [47].

The present study also identified important host-related risk factors. Animals with a body condition score of 2 were more susceptible to fascioliasis than animals with a body condition score of 3. Poor body condition is often linked to inadequate nutrition and weakened immune function, which can reduce the animal’s ability to resist parasitic infections [48]. Additionally, Friesian and crossbred cattle had higher odds of infection compared to local breeds. This likely reflects the better adaptation of local breeds to endemic parasitic pressures, while exotic and crossbred animals are generally considered less resistant to helminth infections [49].

### Snail vectors, cercarial shedding, and environmental suitability

The current findings show that snail intermediate hosts for fascioliasis and schistosomiasis are found across the studied wetlands. However, infected snails were only detected at a few sites, with three infected snails found in the Kajevuba wetland. *B. pfeifferi* and *B. sudanica* were linked to *S. mansoni*, while one *Lymnaea natalensis* was infected with *Fasciola* spp. The overall rate of snails shedding trematode cercariae was 1.4%. This is lower than what has been reported along the Mara River in Kenya and Tanzania [42]; however, even a small percentage of infected snails can maintain transmission because infected snails can stay infectious for life and may release large numbers of cercariae [8]. Snails that did not shed cercariae might have been uninfected, affected by environmental factors, or harboring immature parasite stages that were not yet ready for shedding [8, 50]. These results highlight the need for broader studies across different ecological zones and seasons.

The environmental data further indicate that water chemistry may influence transmission suitability. Parameters such as pH, electrical conductivity, and redox potential appear relevant to the presence and abundance of coexisting *Bulinus* spp. and *Biomphalaria* spp. Wetlands, irrigation channels, and muddy semi-oxygenated soils are known to provide favorable conditions for *Fasciola* transmission and the survival of its intermediate host snails [51, 52]. In this study, the mean pH (7.3), temperature (29.1°C), electrical conductivity (234.9), NaCl concentration (114.8), and redox potential (-29.75) generally align with conditions suitable for the survival of *Fasciola* and its intermediate host snails. These snails tend to prefer neutral to slightly alkaline pH and moderate redox conditions [23]. Similarly, *Schistosoma* transmission is promoted in stable freshwater environments with neutral to alkaline pH and moderate to high redox potential [23]. Thus, environmental conditions in the study area may directly affect infection risk by supporting both snail survival and parasite development.

### Absence of detected human infection and possible explanations

The absence of *Fasciola* and *Schistosoma* eggs among sampled human participants does not necessarily mean there is no disease in the study area. Previous studies in Rwanda have documented *Fasciola* infection in livestock [13, 30] and *S. mansoni* and *S. haematobium* infections in humans [23, 28]. The 0% prevalence observed in this study aligns with findings from similar settings, such as a community-based study in Malawi that also reported 0% prevalence of human fascioliasis among 538 participants [53]. Overall, these results may suggest a truly low disease burden in some populations, but they could also be influenced by methodological and sampling limitations, including small sample size, health facility-based recruitment, effective deworming programs, parasite biology, and imperfect diagnostic sensitivity.

One explanation is that the identified transmission hotspots, especially rice-growing wetlands, were near health facilities. However, few participants attending these facilities were likely rice workers or people with intense wetland exposure. Higher rates of human fascioliasis and schistosomiasis are more often reported in rural farming communities where humans and livestock share water sources and where raw or undercooked freshwater plants may be eaten [42], conditions that might not fully reflect the habits of the sampled population. Additionally, previous studies have shown that stool egg detection is often more successful in community-based surveys than in health facility-based sampling [54]. This is partly because infected patients may visit health facilities during the acute phase, usually 1-3 weeks after infection, when eggs are not yet present in stool [55]. Egg shedding generally begins later, around 8-13 weeks after infection, often when individuals are asymptomatic [22]. Therefore, community-based sampling around hotspots like farms and schools is more likely to give a more accurate estimate of the actual burden of fascioliasis and schistosomiasis [54]. Moreover, symptoms such as abdominal pain, hepatomegaly, and diarrhea are nonspecific and do not reliably identify infected individuals.

A second explanation relates to the biology of *Schistosoma*. Eggs may hatch quickly after contact with freshwater, which can lower the chances of recovery in some field conditions, and the human participants in this study were mainly adults, while schistosomiasis is often more prevalent in children [4]. Human trematode transmission is heavily affected by behaviors such as open urination, defecation, livestock grazing, farming, and swimming in contaminated water bodies [51].

A third possible explanation is the impact of ongoing control efforts. Rwanda has been running a control program since 2008, which includes routine deworming and mass administration of praziquantel, albendazole, and mebendazole in high-risk populations [23]. These measures may have lowered human infection rates in the current setting. However, other epidemiological studies still report persistent *Schistosoma* transmission and the emergence of new foci in Rwanda [23, 25, 29]. Therefore, the lack of detected human cases in this One Health study should not be seen as proof that transmission has completely stopped.

Finally, diagnostic limitations should be taken into account. The current study employed Flukefinder® along with direct microscopy to enhance detection; however, both methods have reduced sensitivity in cases of light infections. Although Flukefinder® is effective at detecting the large, heavy eggs of *Fasciola*, both techniques may miss infections when egg counts are low [38]. Additional research using rapid serological or antigen-detection assays combined with molecular methods would help clarify the true prevalence of human fascioliasis and schistosomiasis in the region.

### Implications for integrated control under a One Health framework

Detecting *Fasciola* and *Schistosoma* in livestock, along with identifying snails shedding cercariae, signals an ongoing risk of exposure for humans, especially those working in or near rice farms. These findings support the need for integrated, multisectoral control strategies within a One Health framework. Such strategies should include regular livestock deworming, water, sanitation, and hygiene interventions for humans, and targeted snail control [1]. Regular livestock deworming can reduce reservoir infections and decrease environmental contamination [56]. Snail control through habitat management, combined with molluscicides when appropriate, remains one of the most effective ways to interrupt the transmission cycle of fascioliasis and schistosomiasis [57]. Additionally, expanding access to safe water, reducing open urination and defecation, and improving hygiene practices through WASH programs can lower snail infection rates and consequently decrease transmission to both animals and humans [58].

### Study limitations and interpretation of the findings

Some limitations should be considered when interpreting these results. The deworming history of slaughtered livestock could not be traced because livestock owners and other stakeholders in the meat value chain often lacked reliable records. The sedimentation method used in livestock may also have missed some infections due to parasite biology; for example, *Schistosoma* eggs may hatch within 20 min after exposure to freshwater at ambient temperature. In the human component, sampling patients at health facilities during symptomatic presentation likely underestimated infections because egg shedding usually occurs later during asymptomatic stages. These factors may have reduced the detection sensitivity and led to an underestimation of the true burden.

### Localized hotspot and transmission risk in Nduba sector

An especially notable finding of this One Health study was the close spatial connection between infected farm livestock and snails shedding *Fasciola* and *Schistosoma* cercariae in Kajevuba wetland, Shango village, Nduba sector. Snail sampling locations were within or near *Fasciola*-infected farms (Figure 2). One *L. natalensis* snail released 21 *Fasciola cercariae*, one *B. sudanica* released one *Fasciola* cercaria, and one *B. pfeifferi* released one *Schistosoma* cercaria. This localized overlap between infected livestock and infected snail hosts strongly indicates active transmission potential and emphasizes the risk of ongoing exposure, maintenance, and persistence of fascioliasis and schistosomiasis in Nduba sector.

### Contribution of the present study

To the best of our knowledge, this One Health investigation is the first study in Rwanda to simultaneously sample human participants, livestock, and freshwater snails, along with assessing environmental water parameters and epidemiological risk factors. This integrated approach offers a comprehensive One Health perspective on the transmission of fascioliasis and schistosomiasis in peri-urban ecosystems. The finding of infected snail intermediate hosts in areas with high human activity highlights potential transmission hotspots and demonstrates the epidemiological connection between snail habitats, livestock grazing areas, and shared water sources.

Previous studies in Rwanda have mainly focused on human schistosomiasis [26, 28] or fascioliasis in livestock based on farm or abattoir surveys [13, 30], without simultaneously investigating the human–livestock–snail interface. Therefore, this study provides new insights into cross-species transmission dynamics and highlights the importance of integrated surveillance for snail-borne trematode infections within the One Health framework. These findings establish baseline data that can support the development of coordinated control strategies targeting both animal and human health in high-risk environments.

### Strengths and limitations of the study

A major strength of this study is its integrated One Health approach, which includes livestock, humans, and snail intermediate hosts within the same ecological settings. This multidisciplinary design enables the identification of localized transmission hotspots and offers valuable information for policymakers to prioritize peri-urban wetlands and agricultural areas for targeted control efforts, such as vector control, livestock deworming, and water, sanitation, and hygiene initiatives aimed at reducing *Fasciola* and *Schistosoma* infections.

However, several limitations should be considered when interpreting the findings. First, the number of human participants included in the study was relatively small, which may have reduced the likelihood of detecting positive cases of schistosomiasis or fascioliasis. Second, sampling was conducted over a limited period during the wet season, which may not fully reflect seasonal variations in snail density, parasite transmission, or the potential effects of climate variability on disease occurrence. Third, identification of snail species was based on shell morphology using standard taxonomic keys, which could lead to misclassification of closely related species. Therefore, more precise molecular techniques should be used in future studies to improve species identification.

Additionally, molecular diagnostic methods would be useful for distinguishing zoonotic species such as *Fasciola gigantica* and *Fasciola hepatica*, as well as for differentiating *S. mansoni* from *Schistosoma bovis* and other related species [59]. Implementing these methods would enable a more precise understanding of transmission pathways and host–parasite relationships, thereby enhancing future One Health surveillance and control programs.

## CONCLUSION

This One Health study showed the ongoing presence of *Fasciola* and *Schistosoma* infections in peri-urban areas of Kigali City, Rwanda, by investigating livestock, freshwater snails, and human participants. Coprological and postmortem exams revealed a high rate of fascioliasis in slaughtered and farm animals, with overall infection rates over 30% in slaughtered animals and more than 40% in live farm animals, while schistosomiasis was less common. Additionally, infected snail intermediate hosts were found in wetlands used for livestock grazing and rice farming, confirming active transmission zones. Although no human cases were identified during the survey, the coexistence of infected livestock and cercariae-shedding snails in the same environments indicates an ongoing risk of zoonotic transmission in peri-urban areas.

These findings emphasize the importance of adopting an integrated One Health approach to control snail-borne trematode infections. Regular livestock deworming, better grazing management, and protecting water sources from contamination are crucial to lowering environmental parasite levels. Additionally, strengthening WASH practices, along with health education and ongoing mass drug administration programs, can help prevent human infections. Environmental management of wetlands and targeted snail control measures should also be considered in high-risk areas, especially in rice-growing ecosystems where livestock, humans, and snail hosts often interact.

The current study provides the first comprehensive baseline data in Rwanda connecting livestock infections, snail intermediate hosts, and environmental factors within the same transmission system. Future research should involve larger human populations, longer seasonal sampling, and broader ecological zones to better understand temporal and geographic variations in transmission. The use of molecular diagnostic tools is also advised to precisely identify *Fasciola* and *Schistosoma* species and to clarify zoonotic transmission pathways. These investigations will enhance risk assessment and aid in developing more effective control programs.

In conclusion, the presence of infected livestock, suitable snail hosts, and favorable environmental conditions confirm that fascioliasis and schistosomiasis are still endemic in peri-urban Rwanda. Integrated surveillance and multisectoral control strategies based on the One Health approach are necessary to reduce transmission, safeguard livestock productivity, and lessen the public health impact of these NTD.

## DATA AVAILABILITY

The supplementary data can be made available from the corresponding author upon request.

## AUTHORS’ CONTRIBUTIONS

JBN, PN, and MN: Conceptualization. MN, PN, EAH, and ASL: Methodology. JBN, MN, ET, SI, JH, VM, JDB, MT, and ES: Investigation and data collection. JBN and EAH: Data analysis and interpretation. JBN: Original draft preparation. JBN, EAH, and ASL: Writing and editing. JBN, MN, ET, JDB, PN, EAH, and ASL: Review. MN and ASL: Supervision. ASL, MN, JBN, PN, and EAH: Funding acquisition. All authors have read and approved the final manuscript.
